# A Review of the Challenges Facing Global Commercialization of the Artificial Meat Industry

**DOI:** 10.3390/foods11223609

**Published:** 2022-11-12

**Authors:** Weijun Liu, Zhipeng Hao, Wojciech J. Florkowski, Linhai Wu, Zhengyong Yang

**Affiliations:** 1 College of Economics and Management, Shanghai Ocean University, 999 Huchenghuan Road, Shanghai 201306, China; 2 Shanghai Social Survey Center, Shanghai Ocean University Branch, 999 Huchenghuan Road, Shanghai 201306, China; 3 Department of Agricultural & Applied Economics, University of Georgia, 1109 Experiment Street, 212 Stuckey, Griffin, GA 30223-1797, USA; 4 Institute of Food Safety Risk Management, Jiangnan University, Wuxi 214122, China

**Keywords:** artificial meat, technology, market, risk, regulatory system, review

## Abstract

The sustained growth of global meat consumption incentivized the development of the meat substitute industry. However, long-term global commercialization of meat substitutes faces challenges that arise from technological innovation, limited consumer awareness, and an imperfect regulatory environment. Many important questions require urgent answers. This paper presents a review of issues affecting meat substitute manufacturing and marketing, and helps to bridge important gaps which appear in the literature. To date, global research on meat substitutes focuses mainly on technology enhancement, cost reduction, and commercialization with a few studies focused on a regulatory perspective. Furthermore, the studies on meat substitute effects on environmental pollution reduction, safety, and ethical risk perception are particularly important. A review of these trends leads to conclusions which anticipate the development of a much broader market for the meat substitute industry over the long term, the gradual discovery of solutions to technical obstacles, upgraded manufacturing, the persistent perception of ethical risk and its influence on consumer willingness to accept meat substitutes, and the urgent need for constructing an effective meat substitute regulatory system.

## 1. Introduction

Rising incomes and social development induced rapid growth in the global consumption of meat and meat products. However, scarcity of resources, outbreaks of animal epidemics (e.g., swine flu), and natural disasters (e.g., typhoons) disrupt the supply of meat. In this paper, artificial meat refers to meat substitutes manufactured using technology converting raw materials, such as plant protein and animal cells, to eliminate the shortcomings of traditional meat protein products. At present, global meat substitutes mainly include plant-based meat (PM) and cultured meat (CM) substitutes [1]. While many researchers regard PM or CM meat substitutes as new food products, few define them as artificial meat products [2,3,4]. However, from the perspective of meat alternatives, they are highly similar and comparable to meat in terms of functional attributes [1].

In recent years, food companies have invested in artificial meat research and development and expect a rapid expansion in retail and food service sales. However, the global commercialization of artificial meat is facing technological innovation challenges, lack of consumer awareness, and inadequate regulation [5]. At the same time, there are several important questions requiring answers and timely solutions that support sustained purchasing and consumption of artificial meat. Among them is the issue of artificial meat safety. Does artificial meat carry ethical and technical hazards? Furthermore, would such hazards negatively affect the sales of artificial meat?

This paper examines four key and intertwined challenges facing the global PM and CM substitute industry: technology, commercialization, hazards, and regulatory oversight. The study conducts a review of relevant published studies. The four subject areas pertaining to the artificial meat sector, limited to PM and CM meat substitutes, are reviewed, specifically: (1) the concept and development process of artificial meat; (2) the technical and market trials in developing the global artificial meat industry; (3) safety hazards faced by the artificial meat industry in the short and long term; (4) and review of regulatory status.

## 2. The Development of Artificial Meat

Growing incomes and changing preferences of the global population increased the demand for protein. Animal protein is the main source satisfying this demand, and its rate of production is expected to grow in the foreseeable future [6,7]. Farmland and labor limitations restrict animal husbandry, reaching a saturation point in recent years [8]. Food producers are required to assure animal welfare and protect land and biological resources [2], making it difficult to increase meat production and supply in the short term.

Meanwhile, COVID-19, African swine fever (ASF), and other outbreaks have already had an impact on the production and supply of global meat products. Traditional animal husbandry, meat production, and processing methods have a negative impact on the environment, health, and animal welfare. Meat substitutes, PM or CM, offer opportunities to reduce the environmental burden of livestock production by using less land and emitting less GHGs [9]. The production of meat substitutes requires less energy overall, and therefore has the potential to address a major global growth constraint.

Meat products not only exact high costs on the environment [10], but also affect public health. Excessive consumption of meat may cause obesity and compromise consumer health. Consumers emphasis on dietary protein [1] and the risk of supply shortages provide opportunities for the development of artificial meat [11]. The development of CM products has been a long process since British Prime Minister Churchill first put forward the concept of CM substitute in 1931 [12]. In 2013, Professor Mark Post finally made the concept a reality [13]. Professor Zhou Guanghong of Nanjing Agricultural University of China cultivated the first cultured meat (CM) in China in 2019 [14]. Meat substitutes offer a solution to the consumer’s desire to eat meat while protecting global food security, and assuring protein supply in the future [5,15].

The development of the artificial meat industry is associated with several hazards. Technical hazards result from unproven technology [16]. New ingredients, especially in the case of CM, present biological hazards [17]. Unclear product positioning and a lack of regulations yields ethical risk [18]. Distrust affects cooperative relationships [19,20] between consumers and artificial meat producers. Therefore, regardless of short-term technical difficulties, an effective regulatory system is necessary for long-term safety [5].

## 3. Types and Technology of Artificial Meat

Artificial meat is a meat substitute [5,15]. The use of raw materials and technology distinguishes artificial meat types as PM and CM products [21,22,23]. Plant-based meat substitutes are also termed “plant-based meat”, “vegan meat”, and “simulated meat”. PM substitutes are mostly made from soybeans. The high moisture extrusion of protein concentrate and water mixtures promotes the development of fiber intermediates to imitate the texture and firmness of meat, and high humidity extrusion technology is used to make meat imitation products retain fragrance to imitate meat flavor [24,25]. Additionally, PM substitutes help overcome resource constraints and limit waste disposal [26,27].

The CM substitute is also known as “synthetic meat”, “in vitro meat”, and “cultivated meat”, but “cultured meat” is most widely accepted and used in the industry. Real-world research into CM substitutes was initiated by NASA [21]. NASA used skeletal muscle tissue engineering, stem cells, cell co-culture and tissue culture to obtain meat culture in vitro [28]. Those efforts aimed to fully mimic the physical sensation of meat, such as visual appearance, smell, texture, and taste [28]. CM substitutes have the potential to replace, at least partially, livestock production and increase animal protein availability to consumers.

Meat substitutes using plants as raw materials have used bean curd, bean skin, and other bean products [29]. Traditional foods such as bean curd and other bean products can be considered as the concept prototype of PM products. Vegetable protein meat analogues have a long history, and are not a new food category [30]. However, PM products constitute a new category, quite different from traditional vegetable protein products, and are produced using a different technology. PM products have a fiber-like structure. The structure imitates the texture and taste of real meat. Yeo and Kim (2020) [31] applied 3D technology that has already been widely used in food production to the manufacture of PM products. The application of electrospinning [31], extrusion technology [26], and 3D printing technology [24,32] transitioned the PM substitute from a concept to reality. 

CM substitutes are in a critical stage of technological breakthrough and commercial application development [33]. The production of CM products can be divided into three stages. The first requires identifying the cell acquisition source. CM products are produced by culturing animal muscle stem cells, but there is a hazard associated with inadequate source cell and culture environment safety [34]. Stem cell acquisition [35] and muscle stem cell maintenance [36,37] solved those problems [23,38,39]. Second, the cell culture and myoblast technology involve a serum-free medium [37,40,41], seed cell mass culture (i.e., microcarrier suspension culture, immobilized culture, or aggregate suspension culture) [39,40,42,43,44], and large-scale differentiation of seed cells into myotubes [45,46,47,48] to gradually improve the culture environment. Finally, through commercial processing and scaling-up, it is possible to produce CM substitutes on a large-scale [39,40]. CM substitute manufacturing involves a co-culture of myoblasts and fibroblasts as the main techniques [49].

While CM production is supported by a large number of new technologies, technical difficulties in cell source acquisition and cell culture have been overcome, very gradually, in bringing CM product to reality. Imperfect technology and manufacturing costs are factors keeping CM products still in the laboratory stage, making it difficult to scale-up production in the short term [35]. Currently, CM technology faces challenges in the preparation of stem cells, optimization of culture conditions, and development of a cost-effective and efficient culture medium [50]. For example, the effective culture of CM products depends on the culture source and composition of the culture medium [23]. A simple and efficient method to generate skeletal muscle cells from mouse skin has been developed providing a source of cell culture [47]. Improvements in CM production capacity and cost reduction are needed to take advantage of market demand. In the UK, an independent technology innovation center and founding member of the UK Government’s High Value Manufacturing Catapult (CPI), has announced the commencement of a project in collaboration with 3D Bio-Tissues Ltd. (Miami, FL, USA) (3DBT) to develop an improved growth media for culturing meat cells in a lab environment. The project aims to increase CM yields and remove the need for animal-derived products, making cellular agriculture as sustainable and economic as possible [51].

## 4. Markets, Consumers, and Artificial Meat

### 4.1. Consumer Expectations Regarding Meat Substitutes

The demand for animal protein is projected to require that nearly two-thirds of farmland be used for animal husbandry by 2050 [52]. The remaining one-third may be insufficient to meet demand for plant-based food production [3]. Consumers have diverse opinions on whether trends in meat consumption needs to be changed [9]. Traditional meat meets basic requirements regarding taste, flavor, nutrition, and cost. Consumers are willing to choose meat substitutes to offset the negative effects of meat consumption [53]. With the improvement of living standards, consumer expectations will be higher for meat substitute attributes [9,54].

Lynch & Pierrehumbert (2019) [55] believe that meat substitutes, as a high-tech replacement for traditional meat, must be competitive in the market by being affordable for consumers and profitable for manufacturers. The commercialization of products is greatly enhanced by consumer acceptance [56,57]. The number of PM products has been growing on the global protein food market [29]. The growth reflects the innovation of meat substitute manufacturers [54]. The number of enterprises joining the PM product market increases year by year, and the types of PM products sold on the market have expanded [29]. Since 2015, the number of meat substitute products (mostly PM products) has more than quadrupled (an increase of 429%). PM products that mimic the characteristics of traditional meat products have already found a place in the protein food market [29]. There are differences across countries and regions in perception and acceptance of meat substitutes [18]. Consumers in the United States and Australia have a high acceptance of PM products, but some still express concerns about the limited variety [58,59].

Several companies, such as Impossible Foods [60] and Beyond Meat [61] have begun PM substitute production. Beyond Meat issued an IPO in 2019 [62] and further promoted technical research into PM substitutes. According to Businesslive’s report on 9 December 2020, Nestle is committed to seizing the Chinese market and has launched Nestle PM products. Their Harvest Gourmet series includes six kinds of PM products. Meanwhile, the company planned to launch related food products through the Tmall online platform and the HEMA Xiansheng off-line supermarket in Beijing and Shanghai [63]. 

At present, the ability of either PM or CM products to meet the needs of consumers is progressing at different paces. CM products have not yet entered the market on a large scale. Vainio, Irz & Hartikainen (2018) [64] suggested that information would change the behavioral intention of “meat skeptics”, and that the way in which that information is expressed determines its effectiveness in changing behavior. Bryant & Barnett (2018) [56] posit that with the commercialization of technology, consumers will be more interested in the acceptance of CM products. While German consumers show moderate acceptance of CM products, they also express concerns about the global spread of unregulated CM products [10]. The overwhelming majority of Chinese urban consumers are unacquainted with meat substitutes including CM [4]. Mancini & Antonioli (2019) [65] found that more than half of surveyed Italian consumers (54%) indicated that they would like to try the CM products. Most were young consumers with high educational attainment who were familiar with livestock farming. In a survey of 5586 consumers in German and French speaking areas of Switzerland, the consumption of meat, health awareness, gender, age, and education affected consumer acceptance of meat substitutes [16]. Among Chinese consumers, acceptance largely depends on their trust in the product.

However, there are a few companies that entered the market offering CM products. According to the AFN website on 2 December 2020, the Singapore Food Administration has approved the US start-up Eat Just to sell its laboratory-grown chicken in Singapore, making Singapore the first country in the world to allow the sale of laboratory grown meat. An Israeli start-up company, Future Meat Technologies (FMT, https://future-meat.com/, accessed on 10 January 2021), plans to launch a production line of 100% CM products in 2022, reducing the cost to less than $22 per kilogram [66].

### 4.2. Meat Substitute Taste

Consumer preferences are mainly affected by taste, price, and other factors [56]. Currently, meat substitute products cannot meet consumer expectations regarding taste and price, explaining the guarded consumer response. Large-scale production of PM and CM products with all the characteristics of meat taste and flavor poses a great challenge for manufacturers [23]. PM substitutes strive to achieve a true meat appearance and taste [7]. CM products have yet to meet consumer taste expectations and experience insufficient production capacity contributing to the high cost in the short term [5]. There has been some progress; for example, Yang et al. (2011) [67] studied meat flavoring systems, which promote the production of meat flavor compounds. Volunteers evaluated the CM product obtained from swine muscle stem cells from Professor Zhou Guanghong’s team, concluding that it was consistent with ordinary meat [14]. Aleph Farms, an Israeli start-up, obtained real meat directly from cow cells. Israel’s Prime Minister Benjamin Netanyahu declared the product delicious, guilt-free, and indistinguishable in taste from traditional beef. Netanyahu was the first head of state to taste meat cultivated outside of a cow [68].

To improve the taste and to extend quality guarantee period, food additives have been viewed as important by experts and regulatory agencies. In 2017, the WHO Expert Committee on food additives proposed that the regulation of food additives should be product-specific. It is necessary to screen additives before new products are marketed. To improve the appearance of artificial meat, food additives such as dyes are used to imitate real meat coloring. For example, synthetic hemoglobin is added to artificial meats. However, additives may not be food grade, and can pose a safety hazard [69,70]. The marketing of PM products focuses on the improvement of color and a number of other attributes [23,56,71]. Consumers prefer natural additives to chemical additives in meat substitutes [72].These problems account for the reluctance of consumers to accept meat substitutes. Consequently, market size growth has failed to meet the expectations of industry experts.

### 4.3. Artificial Meat Cost

Production cost is the most significant challenge to meat substitute technology [16]. The high cost of production is one of the main reasons why meat substitutes are slow to be commercialized [73,74]. Although the price of PM products is somewhat higher than that of traditional meat, the price is acceptable to vegetarians and animal welfare advocates, creating a niche market. However, CM products, for the time being, are very expensive. The first cultured meat took about three months to grow, and cost more than $330,000. Relevant experts and scholars believe that the production of CM products is still at a nascent stage [16,23]. The two examples of CM products listed above are more of an exception than a rule. Artificial meat has been suggested as a source of protein to replace animal protein sources.

Affordability is a prerequisite of novelty food product commercialization, and CM production costs result in prohibitively high product prices. Whether CM products are sold in a developing or developed country, they may be a new source of unfairness because they may be accessible only to the well-off. The meat substitute industry still needs to convince consumers of the value of the product in relation to its price, and make the public aware of the benefits the product offers (taste, safety, health), as well as the societal benefits it offers in terms of protection of the environment and food security [2,75].

## 5. Artificial Meat Ethics and Safety

### 5.1. Ethical Considerations

Ethical safety is one of the important factors affecting consumer acceptance [16]. Consumer perception of objects is an emotional response [73]. Whether the incidents of mistreatment of livestock can reduce consumer negative emotions in consuming meat has attracted much attention [76]. Public attention to ethical issues may affect consumer behavior [77] and affect consumption choice and willingness to purchase a product. Currently, public concerns about animal welfare force the meat industry to constantly evaluate its practices in view of such concerns in China [77]. Dilworth et al. (2015) [78] noted that academic studies regarding livestock production ethics generally support PM and CM production from the perspective of animal welfare and environmental safety. However, consumer concerns regarding the ethics of meat substitutes also involve the uncertainty of the attribution of meat substitutes [77].

Many consumers have a range of ethical concerns about meat substitutes. Pliner and Hobden (1992) [79] proposed that consumer acceptance of new food was affected by “new food technology phobia”. The perception of ethical risks involving meat substitutes ought to be vigorously researched. Potential unknown risks are often attached to new foods and technologies [75,80]. Ethical factors also affect consumer acceptance of meat substitute products [81]. First, consumers have expressed doubts about whether manufactured meat substitutes can be eaten, which also includes consideration of the ownership of meat substitutes. Second, with the improvement of food technology, social development, and living standards, consumers have a new psychological standard for the cognition and requirements of meat substitutes [3].

Once cultured beef was publicly eaten in 2013 [82], the concept of CM was transformed into reality. The problem of “how to produce safer, healthier, and more efficient artificial meat” has been solved, and the focus of consumers has shifted to the discussion and evaluation of “whether or not to make artificial meat”. Cruz Hernández et al. (2019) [83], examining plant-based protein, identified consumers health concerns, safety and nutritional characteristics as important factors. Van der Weele & Driessen (2013) [84] see the need to ponder the purpose, feasibility and practicability of the production of CM substitute. Is the purpose of meat substitute to ease world hunger, or to profit the industry? For developing economies, the former purpose is of primary concern, while consumer acceptance of the product blurring the boundaries of morality is ignored [16].

Consumers pay attention to the production mode and ingredients of meat substitutes. Fetal bovine serum (FBS) is one of the main supplements of CM products. Its acquisition method is considered by many to be inhumane, causing some consumers to reject meat substitute products. Mohorčich and Reese (2019) [85] believe that consumer response to CM products may involve concerns about nature and human character, similar to attitudes towards genetically modified foods. Some consumers believe that meat substitutes, especially CM products, are unnatural and may harm human health, and therefore oppose the development of substitutes [56,78,85,86].

The emergence of meat substitutes allows the public to avoid the ethical dilemma of slaughtering animals to provide meat. Meat substitutes address the competition between humans and animals and offers a more humane relationship with livestock animals [87]. To solve world hunger and environmental problems, potential safety hazards need to be rationally addressed through science [73]. The technology involved in the production of meat substitutes should not blur moral boundaries, and should meet ethical requirements.

Understanding information is the foundation of trust. Consumers generally decide whether to trust a product based on their understanding of the technical principles involved in the production process, information confirmed by the label, and the credibility of information sources [88]. Consumers tend to accept authoritative information sources, but tend to focus on the potential long-term negative health effects of CM products in the face of unknown risks [73]. Although the consumption rate of meat substitutes is still low, consumers are becoming increasingly aware of the link between meat consumption and personal, animal, and environmental health problems [89]. The number of respondents in UK who trust alternative protein sources has reached 53.8% [90]. The study of consumer attitudes towards CM products in Belgium, Portugal, and the UK [73] revealed that consumers would consider gaining direct personal benefits from purchasing meat substitutes, but they pay extra attention to the naturalness and wholesomeness of meat substitutes. Such questions regarding quality leads to fear of meat substitutes, and this continues to affect their perception of these products. The trust of consumers will determine the market success of meat substitutes [2,75,91]. A review of relevant studies from various countries can identify misunderstandings among consumers which diminish trust in meat substitutes [10,16,92]. Consumers in some countries have gradually changed their meat consumption patterns due to historical factors (e.g., meat safety crises) [75]. In other countries, the meat substitute industry needs to tackle the negative perceptions of consumers [75,93]. Consumers supported the study of meat substitutes, but the majority believed that most consumers would not buy such products [94]. The uncertain health effects, taste, and opaque production processes of meat substitutes affect, to a varying extent, consumer acceptance [72,95,96].

To address consumer distrust of meat substitutes, it is necessary to gather information which can lead to solutions. In the early stage of development, enterprises or R & D institutions should invite consumer participation to promote the understanding of technology [95] and the food development process [80]. At the same time, relevant government agencies should be responsible for disseminating pertinent scientific information about meat substitutes to improve consumer acceptance and understanding of the product. Consumers’ misgivings regarding ethical hazards call for thoughtful consideration by practitioners, standardization officers, and regulators [97].

### 5.2. Nutrition and Health

Research on consumer preference for meat substitutes is needed [53]. Those concerned about food safety seek products offering safety, nutrition, and quality [75]. Mohorčich and Reese (2019) [85] argued that the concepts of “unnatural” and “unknown” should not be distorted when considering potential long-term consequences for producing meat substitutes. There are still some reservations about whether meat substitutes offer a high-quality protein comparable to meat. A recent study suggests that the safety of “artificial meat” and food safety hazards have not been determined [98]. Bohrer (2019) [29] and Curtain and Grafenauer (2019) [54] state that modern meat substitutes can provide almost the same nutrients as traditional meat products. It is especially important, prior to CM product sales, to conduct credible health and safety inspections and to implement quality control. Extraction techniques have been applied to improve the separation of individual protein components and eliminate dysfunctional phenolic compounds [11]. Consumers are concerned about the potential negative long-term effects of CM substitute on their health [99]. Inhibition of pathogenic and polluting bacteria can improve the safety of meat substitute production and extend the expiration date [100]. The US start-up Eat Just, in the application documents to Singapore to prove that its production of CM products is a stable manufacturing process, indicated that it had constructed more than 20 1200-L bioreactors. At the same time, the company also proved to the Singapore Food Authority that its products meet the existing poultry industry standards, and that the CM products have lower microbial content and are cleaner than traditional animal derived meat [101]. The low microbial content and elimination of the need for space, feed, waste disposal, and bird processing result in reduced risk of environmental pollution. They also conserve natural resources. The process is particularly attractive to an agricultural resource-constrained Singapore.

## 6. Hazards in Meat Substitute Manufacturing

### 6.1. Hazards in PM Manufacturing and Distribution

The stages of PM product manufacturing include planting (raw material acquisition), production, processing, and distribution.

**Figure 1:**
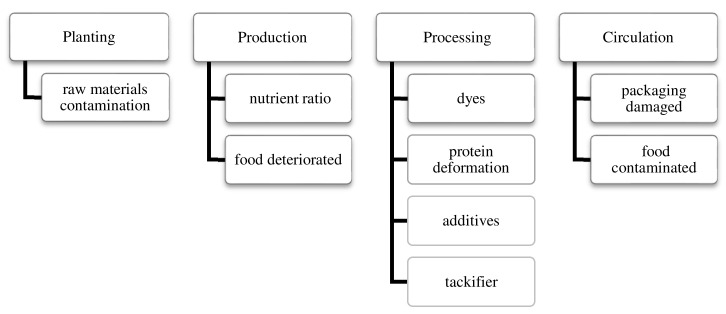
Sources of hazard in the manufacturing and distribution of PM products.

The raw material contamination requires attention during raw material acquisition of the plant protein ingredient (Figure 1). For example, soybean and other plant-based raw materials during the fermentation process may be affected by inadequate control, causing protein damage, and could result in contamination of the fermented material [102,103].

In the production stage, attention must be paid to the proportion of ingredients, preservation of semi-finished products, and specification of ingredients. Incorrect ingredient distribution ratios may lead to differences in nutrient absorption between PM and ordinary meat [41,104]. Additionally, improper operation may lead to CM contamination during fermentation [105,106]. Also, the degree of protein deformation may exceed or be lower than the desired range. At the product distribution stage, there could be problems of package damage and product deterioration [103].

### 6.2. Source of Hazards in CM Manufacturing and Distribution

**Figure 2:**
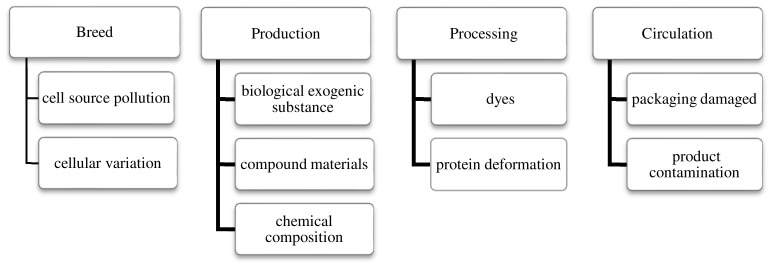
Hazards affecting quality and safety of CM products.

Figure 2 shows possible sources of hazards at different CM product manufacturing and distribution stages. In the cell breeding stage, the key is to qualify the cell source. During the seed cell transformation, the introduction of high-risk biological contaminants is possible, which would cause cell pollution and variation [102].

The production stage must focus on the ingredients and composition. There is uncertainty associated with the compound composition, scaffold material, and mold material [47,107]. There are also possible differences in nutrient absorption between CM and ordinary meat [69,70]. It is necessary to monitor the culture environment, because CM is easily affected by various microorganisms [108,109].

## 7. Regulation of the Artificial Meat Industry

To promote meat substitutes, examination of the status quo of the meat substitute industry under existing policies and endorsement of regulatory changes to local, national and international food systems are urgently needed. The meat substitute industry faces uncertainty, due in part to the regulatory system [110,111]. Policy and regulatory changes will affect the entry of meat substitutes into the market [16]. Feasibility, rationality, value, integrability and sustainability were proposed as criteria to improve the market acceptance of a product and can be applied in the case of the meat substitute [112]. According to the rules and regulations of good cell culture practice (GCCP), it is necessary to develop meat substitute production system [37].

Stephens et al. (2018) [16] believe that meat substitutes should be classified as food rather than a medical product, relaxing regulatory requirement needed for medical applications. In terms of obtaining source cells, muscle stem cells are essential for CM meat substitutes. To determine stem cell high proliferation ability, pluripotent cells (mesenchymal stem cells, etc.) have commonly been studied [113,114,115]. The use of certain cell sources, like the tissue culture of meat, could have crucial ethical implications and impacts on health, and needs to be controlled by new regulations.

### 7.1. Regulation Status

Meat substitute products have not been clearly classified. PM and CM technology differs from that used in production of traditional meat-like items, and their attribution and identification are needed. The standards of CM products differ in various countries and regions. Petetin (2014) [116] concluded that it was not possible to assess the regulations applicable to meat substitutes. To maintain regulatory power over production facilities, regulatory authorities need to classify meat substitute products. In Europe, meat substitute was classified as a new type of food in October 2018. In Australia and New Zealand, cultured meat can comply with existing food standards and specifications after obtaining approval before entering the market [117]. In China, the regulatory system of meat substitute is still in the early stage of exploration, and it is necessary to promote the scientific development of technology, standards, and supervision of CM substitutes. There could be a need to implement different regulatory approaches based whether the facility is categorized as an agricultural or food processing entity. In the United States, the FSIS and FDA proposed in March 2019 to jointly supervise manufacturing of meat substitute products originating from livestock and poultry cell lines, and divide the regulatory content [118].

From a public opinion and trade policy perspective, regulations are largely viewed as inadequate [77]. The assessment and management of CM products should be independent from PM product rules and include CM product manufacturing [16]. The development of a new type of food creates many problems in the supervision of meat substitute production, and imperfect supervision can be detrimental to industrial development.

### 7.2. Regulatory Framework

In order to deal with technical standards, standardization, manufacturing hazards, biological safety, and ethical issues, the regulatory system of the meat substitute industry should be constructed with reference to a common international framework. Although the composition and priority development areas of food control systems vary from country to country, most of them include a regulatory system, management system, scientific support system, an information education exchange, and a training system. According to Guidelines for Strengthening National Food Control Systems [119], the implementation framework of a supervisory system for the meat substitute industry should involve the construction of standards, a regulatory system, and a management system.

Standards improve consumer awareness and prevent businesses from using banned substances. Also, the guidelines for labeling meat substitute products are chaotic. It is necessary to ensure that the label content is consistent with the product [95,120]. A label allows consumers to distinguish new products from traditional meat products, and supports meat substitute public acceptance. Through multi-channel and multi-angle publicity, consumers can gain an understanding of meat substitutes, further improving product acceptance.

A regulatory system forms the basis of the food safety system and improves the mandatory laws and regulations related to food. The legislation of meat substitute product regulations should provide a high level of health assurance [119]. Moreover, guidelines will assure consistency and legal rigor, transparency and independent risk assessment, and help in risk management and risk communication. Guidelines should include preventive provisions, provisions on consumer rights and interests, and provisions on traceability and recall. Additionally, the guidelines should explicitly stipulate the responsibility of food producers and manufacturers for product quality and safety. Finally, the guidelines should stipulate the obligation to ensure that the meat substitute products are sold and distributed safely and fairly. The law should be recognized by governments and through international obligations, especially trade-related, ensuring transparency and uninterrupted access to new information.

Different countries have different regulatory methods for meat substitutes, but the development of the meat substitute industry is complex, and a “main authority” mode should be advocated in which departments with expertise in this field should supervise all other departments [16]. In terms of legal and regulatory system construction, the EU provides framework provisions for member states, but the new food regulations need to be improved. The legal construction in the United States is more detailed. In general, there are two ways for food management agencies in Europe and the United States to deal with emerging food products. One is to compare the new products with the existing products that have been tested and adopt the already existing safety management regulations; the other is for a new food to be classified as such, in which case new regulations should be formulated.

The cell sources, culture methods, labeling, quality management, and hygiene of CM products have stimulated the establishment of appropriate regulations and a regulatory framework [118]. To address management issues, the U.S. Department of Public Health, together with the Food and Drug Administration (HHS-FDA) and the USDA Food Safety Inspection Service (USDA-FSIS) announced on 7 March 2019 that they would jointly supervise the production of meat substitute from livestock and poultry cell lines. Among them, FDA supervises cell collection, the cell bank, and cell differentiation and growth, while FSIS is responsible for the follow-up supervision of cell collection [118].

## 8. Conclusions

Since the 1990s, the world’s meat consumption has increased rapidly, providing an opportunity for the development of the meat substitute industry. In recent years, artificial food and insect food have developed rapidly in the world food market, while in vitro meat production technology has improved continuously. Technology improvement, cost reduction, regulations, safety, and consumer acceptance are the main factors affecting the development of the meat substitute industry. However, consumer concerns pose the most important long-term challenge to the meat substitute industry.

According to the OECD-FAO forecast, world meat consumption will continue to grow in the future. The supply gap of meat products for China, for example, may exceed 38 million tons in 2030. If the penetration rate of meat substitutes reaches 5%, the value of the meat substitute market will reach $100 billion. The COVID-19 epidemic, among other factors, resulted in a pork shortage. The discrepancy between protein demand and meat supply could lead to a rapid growth of the “meat substitute” market. With consumer acceptance increasing and resistance to meat substitute marketization weakening, an opening for meat substitutes is being created.

Currently, there is room for meat substitute technology improvement. First, there is a need to efficiently simulate animal muscle tissue growth and scale up production in bioreactors. Secondly, the taste of meat substitute products still does not match that of traditional animal meat. If the meat substitute taste does not resemble that of real meat, the meat substitute can only be considered a novelty and possibly assure food security, but cannot replace real meat. Third, the technology of in vitro meat production (culture of myoblasts and fibroblasts) has yet to be applied on a large scale.

Experts across the world are committed to providing breakthrough solutions to improve the environmental effects, taste, commercialization and consumer acceptance of meat substitutes. European and American investors allocated substantial resources to develop efficient and safe cell culture technology. The development of the meat substitute industry in Europe and the United States shows that demand preferences in the early period of meat substitute development have changed greatly. Demand drivers stimulate the research and industrialization of meat substitutes, gradually overcoming the technical and technological problems.

The ethical and social problems arising from the production and consumption of meat substitutes are both novel and complex. The attributes, ethical and moral problems, psychological acceptance, and aversion of consumers of meat substitute products need to be explored and resolved. In the short term, it is unrealistic to expect to eliminate consumer concerns about meat substitutes, or to resolve the debate over the morality of the development and application of meat substitute technology. Over time, stakeholders can find the most appropriate way to deal with the science and technology of meat substitutes. An effective regulatory system to manage safety risks of the meat substitute industry is needed. The global climate, lack of environmental protections, and continuous population growth multiply safety risks associated with livestock and meat production. The development of “alternative meat” has become a global trend. A meat substitute may eliminate meat supply shortages, making it worthwhile to develop the technology necessary to establish a sustainable meat alternative. The development of a sustainable meat alternative depends on risk prevention and control, standards, laws and regulations. Dealing with the regulatory issues in a forward-looking manner, the meat substitute industry can contribute to better lives, ensuring safety, while also protecting the environment.

## Data Availability

Data is contained within the article.
